# Elements of metacommunity structure in Amazonian Zygoptera among streams under different spatial scales and environmental conditions

**DOI:** 10.1002/ece3.2849

**Published:** 2017-03-31

**Authors:** Leandro Schlemmer Brasil, Thiago Bernardi Vieira, José Max Barbosa de Oliveira‐Junior, Karina Dias‐Silva, Leandro Juen

**Affiliations:** ^1^ Programa de Pós‐Graduação em ZoologiaUniversidade Federal do ParáParáBrazil; ^2^ Programa de Pós‐Graduação em Biodiversidade e ConservaçãoUniversidade Federal do ParáParáBrazil; ^3^ Instituto de Ciências e Tecnologia das ÁguasUniversidade Federal do Oeste do ParáSantarém, ParáBrazil; ^4^ Instituto Nacional de Pesquisa da Amazônia ‐ ManausAmazonasBrazil; ^5^ Instituto de Ciências BiológicasUniversidade Federal do ParáParáBrazil

**Keywords:** aquatic insect, diversity decline, freshwaters, integrity, landscape

## Abstract

An important aspect of conservation is to understand the founding elements and characteristics of metacommunities in natural environments, and the consequences of anthropogenic disturbance on these patterns. In natural Amazonian environments, the interfluves of the major rivers play an important role in the formation of areas of endemism through the historical isolation of species and the speciation process. We evaluated elements of metacommunity structure for Zygoptera (Insecta: Odonata) sampled in 93 Amazonian streams distributed in two distinct biogeographic regions (areas of endemism). Of sampled streams, 43 were considered to have experienced negligible anthropogenic impacts, and 50 were considered impacted by anthropogenic activities. Our hypothesis was that preserved (“negligible impact”) streams would present a Clementsian pattern, forming clusters of distinct species, reflecting the biogeographic pattern of the two regions, and that anthropogenic streams would present random patterns of metacommunity, due to the loss of more sensitive species and dominance of more tolerant species, which have higher dispersal ability and environmental tolerance. In negligible impact streams, the Clementsian pattern reflected a strong biogeographic pattern, which we discuss considering the areas of endemism of Amazonian rivers. As for communities in human‐impacted streams, a biotic homogenization was evident, in which rare species were suppressed and the most common species had become hyper‐dominant. Understanding the mechanisms that trigger changes in metacommunities is an important issue for conservation, because they can help create mitigation measures for the impacts of anthropogenic activities on biological communities, and so should be expanded to studies using other taxonomic groups in both tropical and temperate systems, and, wherever possible, at multiple spatial scales.

## 1 Introduction

A fundamental goal of community ecology is to understand patterns of species distributions (Sutherland et al., 2013). Species distributions at the metacommunity scale result from the interplay between spatial and environmental processes, and biotic interactions (Soberón, 2007). These conditions are discussed in four mechanisms of metacommunities structure: (1) patch dynamics, (2) neutral effects, (3) species sorting, and (4) mass effects, which may act either in isolation or in combination (Leibold et al., 2004), on metacommunity structures, based on their patterns of coherence, species turnover, and boundary clumping (Leibold & Mikkelson, 2002).

Metacommunities are made up of sets of communities potentially connected through the dispersal of species (Wilson 1992). In the context of metacommunities in the Amazon biome, the distribution of some organisms, such as monkeys (Wallace 1854), birds (Ribas et al. 2011), and Zygoptera (Juen and de Marco, 2012), is determined by major rivers, which have acted historically as geographic barriers to migration, limiting the dispersal capacity of many species. Accordingly, there are eight areas of endemism, each bounded by large Amazonian rivers: Guiana (region of interfluve between the Amazon and Negro Rivers), Imeri (Negro and Solimões Rivers), Napo (Solimões and Napo Rivers), Inambari (Solimões and Madeira Rivers), Rondônia (Madeira and Tapajós Rivers), Tapajós (Tapajós and Xingu Rivers), Xingu (Xingu and Tocantins Rivers), and Belém (Tocantins and Amazonas Rivers). Given this biogeographic role of the rivers, each area of endemism probably acts as a distinct metacommunity, with the species being more likely to disperse within an area of endemism than between different areas of endemism.

However, at small spatial scales, environmental conditions of the streams are among the most important mechanisms determining community structure (Monteiro‐Júnior, Juen, & Hamada, 2014; Oliveira‐Junior et al., 2015), as the presence or absence of species will depend on the prevailing conditions (species sorting) (Van der Gucht et al., 2007). Given this, the species composition of a community will be determined principally by environmental filters—Hutchinson's (1959) niche concept—rather than dispersal ability (Leibold et al., 2004). In the mass effect perspective, both regional and local assembly processes play a role important in structuring communities (Amarasekare, 2000). Predictions change if dispersal plays a role in structuring communities. This is because populations will tend to be larger in more appropriate habitat patches, and due to the homogenizing effect of dispersal, communities connected by dispersal should be functionally similar to each other (Altermatt, 2013). Therefore, metacommunities should be influenced by both dispersal among sites and environmental conditions (Heino, Melo, et al., 2015). The patch dynamics approach considers patches with identical conditions, in which local species diversity is determined by dispersal, colonization, and extinctions (Pickett & Thompson, 1978); the neutral perspective assumes that at a given trophic level, species are equivalent in birth, death, dispersal, and speciation rates (Hubbell, 2001). These mechanisms are especially important on a regional scale for species distribution patterns (Cottenie, 2005).

**Figure 1:**
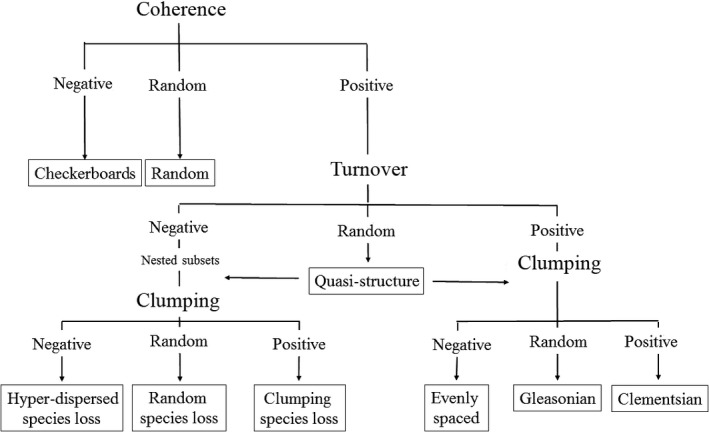
Theoretical framework of the analytical method of elements of metacommunity structure. Modified from Presley et al. (2010)

From the processes mentioned above (species sorting, environmental filters, mass effect, and neutral concept) arise patterns in the distribution of species at the metacommunity level. To analyze these patterns, an analytical routine based on null models has been developed, which distinguishes six idealized “metacommunity structures” (Leibold & Mikkelson, 2002; Presley, Higgins, & Willig, 2010): (1) checkerboard— the distribution of species is influenced primarily by biotic interactions, such as competitive exclusion or facilitation (Diamond & Diamond, 1975); (2) nested—the regional set of species is formed by a series of subsets nested over a spatial continuum (Patterson & Atmar, 1986), which may be related to the environmental conditions of the habitats and/or the intrinsic characteristics of the species, such as their dispersal capacity or tolerance environmental alterations (Heino, Mykrä, & Muotka, 2009); (3) Clementsian—this pattern reflects the effect of biogeographic processes and barriers, leading to the formation of discrete communities within the landscape (Clements, 1916); (4) Gleasonian—communities are structured along some gradient, but species respond to this gradient independently (Gleason, 1926); (5) uniform spacing—continuous gradients formed by the progressive turnover of species within the environment (Tilman, 1982); and (6) random—elements of metacommunity structure no different from those expected by chance (Simberloff, 1983). Additionally, the quasi‐structured pattern covers the cases in which the turnover is equal to that expected by chance, thus reducing the robustness of the nested, Clementsian, Gleasonian, uniformly spaced and random patterns, leaving the metacommunity quasi‐structured (Presley et al., 2010) (Figure 1).

Considering recent ecological factors, the principal environmental filters for Odonata communities are the loss of habitat quality resulting from human activities (e.g., conversion of natural habitats to farmland, urban, or industrial areas) (Monteiro‐Júnior et al., 2014; Oliveira‐Junior et al., 2015). In addition, considering biogeographic historical factors in the Amazon, the formation of endemic areas is an important predictor of zygopteran assemblages at large spatial scales (Juen and de Marco, 2012). As the distribution of Zygoptera is related to both environmental conditions (recent ecological factors) and spatial processes (biogeographic historical factors), we believe that it is an appropriate group for the testing of hypotheses on the patterns and mechanisms that structure metacommunities.

In the present study, we investigated the elements of metacommunity structure of zygopteran species in two Amazonian areas of endemism, which include streams under different levels of anthropogenic influence. Our principal hypothesis was that the metacommunities in preserved (“negligible impact”) sites would present a Clementsian pattern, due to the biogeographic distribution of the species in the areas of endemism. However, these patterns should be modified in the impacted streams, due to homogenization of communities in impacted streams (primarily by agriculture). We also analyzed elements of metacommunity structures at smaller spatial scales, within each area of endemism. This analysis is necessary given that Presley and Willig (2010) found that, in the case of a Clementsian pattern, each distinct geographic block can be identified, and distribution patterns can be re‐analyzed within these blocks, reinforcing the overall perspective on the influence of different processes and mechanisms acting at different spatial scales.

## 2 Material and Methods

### 2.1 Study areas

**Figure 2:**
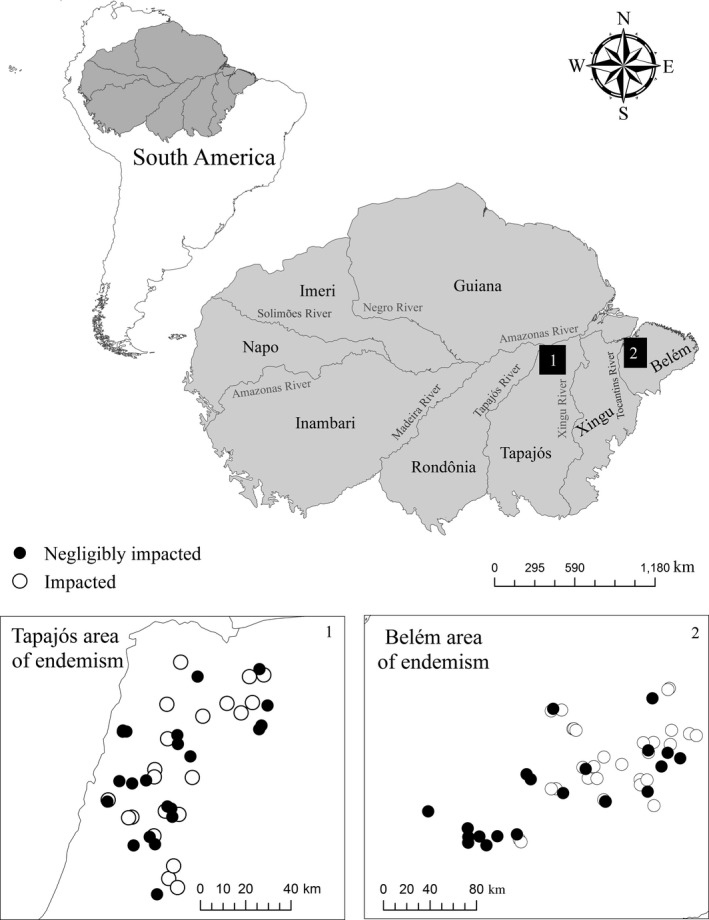
Spatial distribution of the zygopteran communities sampled in the southeastern Amazon basin, with the interfluve of the major rivers shaded gray (areas of endemism). At the left, (1) shows the sites sampled in Santarém, which is located in the Tapajós area of endemism, while at the right, (2) shows the sites sampled in Paragominas, in the Belém area of endemism

We collected adult damselflies (Odonata: Zygoptera) in 93 small streams (no more than 5 m in width and 0.8 m in mean depth), located in eastern Brazilian Amazonia, in the municipalities of Santarém and Belterra in the Tapajós area of endemism (interfluvium between the Tapajós and Xingu Rivers), and the municipality of Paragominas, in the Belém area of endemism (interfluvium between the Tocantins and Amazon Rivers), all in the state of Pará, Brazil (Figure 2). Given the possible influence of isolation by rivers (Wallace 1854), which has been confirmed in Amazonian zygopteran communities (Juen and de Marco, 2012), we considered the Paragominas (located in the Belém area of endemism), and Santarém and Belterra (Tapajós area of endemism) regions, as two distinct biogeographic units in our analyses.

The study region has an *Af*‐type climate, in the Köppen classification (Peel, Finlayson, & Mcmahon, 2007), that is, wet tropical, with short dry periods between June and December (Gardner et al., 2013). In Paragominas (1.9 Mha), mean annual precipitation is 1766 mm, mean annual temperature is 27°C, and relative humidity is 81%. Santarém (1 Mha) has a mean annual precipitation of 1,920 mm, mean temperature of 25°C, and relative humidity of 86% (Gardner et al., 2013).

The natural landscape of the two study regions is formed by equatorial rainforest or *terra firme* forest, although there has been extensive deforestation in many areas (Gardner et al., 2013). The anthropogenic areas are covered mainly by eucalyptus (*Eucalyptus* sp.), teak (*Tectona grandis* L.), or paricá (*Schizolobia parahyba* var. *amazonica* Huber ex Ducke) plantations, cattle pasture, and crops such as rice (*Oryza sativa* L.) and soybean (*Glycine max* L.) (Oliveira‐Junior et al., 2015).

### 2.2 Environmental characteristics

To describe the environmental conditions of the study areas, we measured 12 habitat variables included in the protocol described in Nessimian et al. (2008), which are used to calculate the Habitat Integrity Index (HII). These variables (supporting information) describe land use in the environments adjacent to the riparian zone (variable 1), the environmental conditions of the riparian forest (2–4), and the characteristics of the stream channel (5–12). Each variable is composed of four to six alternatives ranked in accordance with their perceived contribution to habitat integrity. To standardize the measures for analysis, the values were weighted in relation to the maximum value recorded for each item (see equation 1—supporting information). The final index score is the mean value of all the items measured in each habitat (equation 2—supporting information). The result of this procedure is an index that varies from 0 to 1, providing a standardized measure of the integrity of the local conditions found in each habitat (Nessimian et al., 2008).

The HII has proven to be a valuable descriptor of the environmental integrity of Amazonian streams, and when applied to odonate fauna, it has also been shown to be a good predictor of the abundance of individuals and the species richness and composition of these communities (Brasil, Batista, et al., 2014; Brasil, Giehl, et al., 2014; Carvalho, Pinto, Oliveira‐Júnior, & Juen, 2013; Juen, Oliveira‐junior, & Shimano, 2014; Monteiro‐Júnior, Couceiro, Hamada, & Juen, 2013; Monteiro‐Júnior et al., 2014; Oliveira‐Junior et al., 2015). Major alterations, principally in species composition, tend to be observed at streams with integrity values of <0.6 or 0.7. Significant changes tend to be observed in the communities found in habitats with indices lower than this (Brasil, Batista, et al., 2014; Carvalho et al., 2013; Juen et al., 2014; Monteiro‐Júnior et al., 2014; Oliveira‐Junior et al., 2015).

### 2.3 Collection of biological material

We collected specimens in 2010 (Tapajós area of endemism) and 2011 (Belém area of endemism), during the drier part of the year between June and August, when most of the species that inhabit Amazonian streams can be found as adults (Baptista, Dorvillé, Buss, & Nessiamian, 2001; Oliveira‐Junior et al., 2015). At each stream, we demarcated a linear transect of 150 m, along which a trained technician captured all the damselflies spotted during a 60‐min period, using an entomological hand‐net, 40 cm in diameter and 65 cm in length (Oliveira‐Junior et al., 2015). To avoid sampling bias derived from the thermoregulatory behavior of the insects, all sampling was conducted between 10:00 hr and 14:00 hr, when the sunlight reaches the stream bed, and all the different groups—thermal conformers, heliotherms, and endotherms—can be encountered (De Marco, Batista, & Cabette, 2015; De Marco & Resende, 2002; May, 1976).

The specimens were prepared and fixed following the protocol described by Lencioni (2006). Finally, we identified all the specimens collected using taxonomic keys and specialized illustrated guides (Garrison, 1990; Garrison, Ellenrieder, & Louton, 2010; Lencioni, 2005, 2006). Whenever necessary, specimens were sent to the appropriate specialists to resolve their taxonomy. All the specimens were deposited as vouchers in the collection of the Zoology Museum of the Belém campus of the Federal University of Pará, Brazil.

### 2.4 Data analysis

Initially, to define the threshold of habitat integrity along the environmental gradient that divided the sites into two categories (negligibly impacted and impacted), we performed a principal component analysis (PCA) using the 12 environmental variables that make up the HII (Supplementary material 1). Based on this analysis and the findings of previous studies (Brasil, Batista, et al., 2014; Dutra & De Marco, 2015; Juen et al., 2014; Monteiro‐Júnior et al., 2014; Oliveira‐Junior et al., 2015), we defined a threshold of HII = 0.7 to separate the negligibly impacted streams (HII ≥ 0.7) from the impacted (HII < 0.7) streams. While the term “negligibly impacted” is used here to facilitate the comprehension of the results, some of the sites may have suffered a certain degree of anthropogenic impact, but can be considered to be the best conserved sites, given the local context of the region, and adequate for inclusion in the analyses as control sites.

To verify the elements of metacommunity structures, we adopted the approach of Leibold and Mikkelson (2002). The analysis consists of a sequence of tests of the coherence, turnover, and clumping. Coherence is measured by the number of absences found between the occurrences in the matrix, where fewer absences than expected by chance represent a condition of positive coherence, while a greater number than expected by chance represents a negative coherence. Similarly, the turnover is the number of double substitutions in pairs of streams and considered to be positive when this number is larger than the expected value, and negative when the number is lower than expected by random. Clumping or boundary clumping measures the divergence in the limits of species distribution based on Morisita's index, which estimates the clumping of species distributional boundaries (Leibold & Mikkelson, 2002). When the index is higher than one, clumping is positive, and negative when it is lower than one.

We tested the three elements, coherence, turnover and clumping, by determining the probability of accepting the null hypothesis based on 9999 randomizations with a 5% significance level (Leibold et al., 2004; Presley et al., 2010). When coherence is significantly negative, the analysis confirms a checkerboard pattern, but when the null hypothesis is accepted, a random pattern is confirmed. When coherence is significantly positive, the turnover test is implemented (positive or negative than what expected given the null distribution).

When turnover is significantly positive, the clumping is tested, and when this is significantly negative, an evenly spaced pattern is confirmed. When it is random, the pattern is Gleasonian, and Clementsian when significantly positive. In the cases where the turnover is significantly negative, and the clumping is also negative (nested subsets), the data are tested again, and a negative pattern indicates hyper‐dispersed or random species loss, and clumped species loss when positive. When no significant turnover is recorded, and clumping remains positive or negative, a quasi‐structured pattern is identified (Presley et al., 2010).

To identify the elements of metacommunity in zygopteran communities of Amazonian streams according to their level of impact (impacted and negligibly impacted) and biogeographic region (Belém and Tapajós areas of endemism), we divided the data into nine distinct subsets: (1) all the streams, (2) negligibly impacted streams (HII ≥ 0.7), (3) impacted streams (HII < 0.7), (4) all the streams in the Belém area of endemism, (5) all the streams in the Tapajós area of endemism, (6) negligibly impacted streams in the Belém area of endemism, (7) negligibly impacted streams in the Tapajós area of endemism, (8) impacted streams in the Belém area of endemism, and (9) impacted streams in the Tapajós area of endemism. We visualized these patterns graphically through the direct ordination of the communities by the first spatial filter (principal coordinate analysis of neighbor matrices—PCNM1) derived from the geographic coordinates of the study sites (Griffith & Peres‐Neto, 2006). The eigenvector‐based spatial filters (PCNM) from the geographic coordinates of the sites are simple solution to understand spatial patterns. The basic idea is to extract eigenvectors of a distance Euclidean matrix among spatial units (sites) and use these eigenvectors, which describe the spatial structure as a spatial predictor variable (Diniz‐Filho & Bini, 2005).

To test the premise that there are groups of species that reflect the pattern biogeographic regions of the study, we conducted a PERMANOVA (Anderson, 2001; Anderson & Walsh, 2013), with the species composition matrix (presence and absence) including region (Paragominas and Santarém) as a categorical variable. To test whether communities in impacted areas are homogenized compared to communities of negligibly impacted areas, we compared the species composition matrix between negligibly impacted and impacted streams using tests of homogeneity of multivariate dispersion (PERMDISP) (Anderson, & Walsh, 2013).

We ran all the analyses in the R program (Team R, 2013), with the patterns of environmental conditions being tested using a principal component analysis (PCA) run with the “prcomp” function (R stats package), permutational multivariate analyses of variance (PERMANOVA) in the “adonis” function (R vegan package), and permutational analysis of multivariate dispersions (PERMDISP) in the function “betadisper” (R vegan package). To calculate spatial filters, we used the function “PCNM” (R vegan package). The metacommunities were analyzed with the metacom package, using the metacommunity function (Dallas^2014^), and the ordination was produced in the vegan package using the generic function (Oksanen et al., 2013).

## 3 Results

### 3.1 Description of the communities

**Figure 3:**
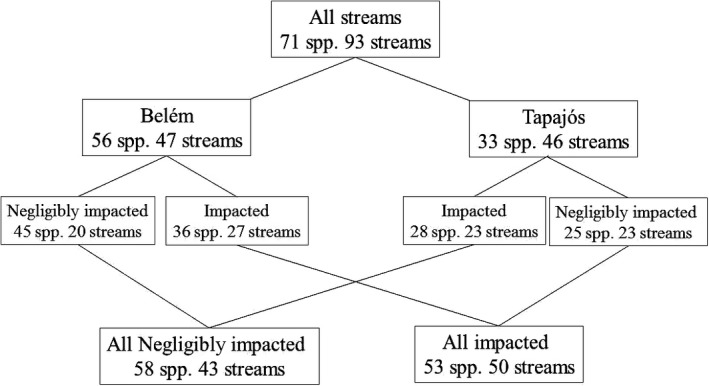
Graphic showing the number of streams (sampling units) and species (spp.) classified by integrity environmental (negligibly impacted and impacted) and biogeographic region (Belém and Tapajós) in eastern Amazonia

We collected 71 species of Zygoptera, of which 57% were found in both negligibly impacted and impacted streams, while 25% were found exclusively in negligibly impacted streams, and 18% only in impacted streams. A quarter (25%) of the species were found in both Belem area of endemism and Tapajos area of endemism, while 21% were exclusive to Tapajos area of endemism, and 54% were exclusive to Belem area of endemism. The negligibly impacted sites were the most species‐rich in both study regions, with the negligibly impacted streams of Belém area of endemism being the richest overall, and the impacted streams of Tapajós area of endemism, the poorest (Figure 3).

### 3.2 Environmental conditions of the streams

**Figure 4:**
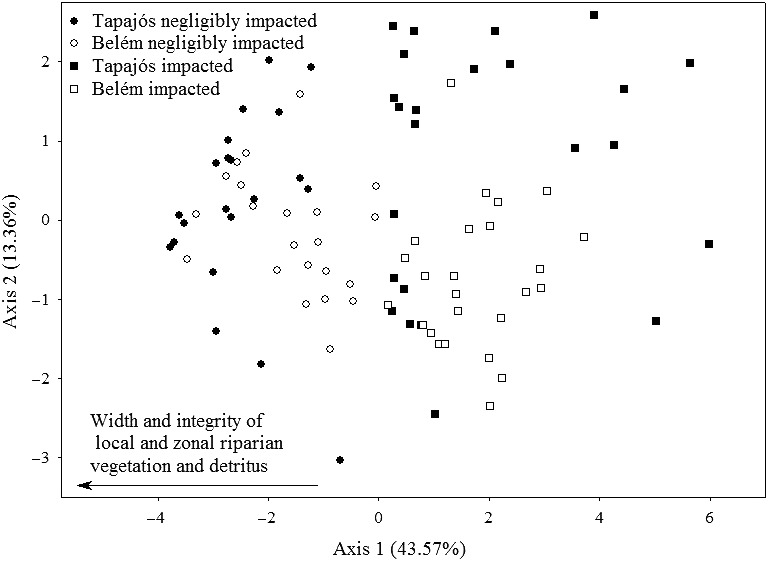
Ordination of the streams based on the 12 variables of environmental integrity used to compose the Habitat Integrity Index of Nessimian et al. (2008)

The ordination of the streams based on their characteristics of environmental integrity revealed a clearly visible separation of the sites, with those of high integrity (HII ≥ 0.7) to the right, and the low integrity streams (HII < 0.7) to the left of the first axis (negligibly impacted and impacted, respectively). The variables that most contributed to this distinction were the structure of surrounding riparian vegetation (less extensive and more degraded in the impacted streams, within a radius of 10 m), and the quantity of debris in the water (higher in impacted streams). These features refer to variables 2, 3, 4, and 12 of the HII (Table 1; Figure 4).

**Table 1:** Correlation between the different variables of the environmental integrity of the streams and the first and second PCA axis (Figure 4). The highest loadings (correlation >70%) are shown in bold

Characteristic	Loadings	
	Axis 1	Axis 2
1‐ Land use pattern beyond the riparian zone	−0.395	0.193
2‐ Width of riparian forest	**−0.876**	0.192
3‐ Completeness of riparian forest	**−0.851**	0.159
4‐ Vegetation of riparian zone within 10 m of channel	**−0.846**	0.167
5‐ Retention devices	−0.674	−0.185
6‐ Channel sediments	−0.612	−0.521
7‐ Bank structure	−0.453	0.668
8‐ Bank undercutting	−0.644	0.232
9‐ Stream bottom	−0.268	−0.602
10‐ Riffles and pools, or meanders	−0.557	−0.295
11‐ Aquatic vegetation	−0.627	−0.460
12‐ Detritus	**−0.799**	0.105

### 3.3 Metacommunity structures

Considering the complete set of communities, the elements of metacommunity structures were quasi‐Clementsian, given that the matrix coherence was significant and positive, although the turnover was not significantly different from what could be expected by random, while the clumping was positive and significant. When only negligibly impacted streams were analyzed, the coherence, turnover, and clumping were all significantly positive, which is consistent with a Clementsian pattern. In the case of the impacted streams, coherence did not differ from random significantly positive, but turnover was random, with values lower than expected and significant clumping, with observed values higher than one, which is consistent with a pattern of clumping species loss (Table 2).

**Table 2:** Metacommunity structures in the zygopteran of negligibly impacted and impacted streams in the Belém area of endemism (BAE) and Tapajós area of endemism (TAE)

	Metacommunity								
	All communities			Negligibly impacted			Impacted		
	All	BAE	TAE	All	BAE	TAE	All	BAE	TAE
Coherence									
*p*	**<.001**	.401	.261	**<.001**	**.001**	.519	**<.001**	.065	.465
Embedded absences	1898	795	603	766	286	231	520	280	178
*Z*	9.073	5.490	1.123	5.634	3.139	0.644	6.313	1.841	0.730
sim. Mean	3550.2	1259	657.879	1152.2	383	244.208	1120.6	348	192
sim. *SD*	182.08	84.631	48.851	68.560	31	20.496	95.139	37.029	19.785
Method	R1	R1	R1	R1	R1	R1	R1	R1	R1
Turnover									
*p*	.226	.560	.436	**<.001**	**<.001**	.417	.995	.261	.427
Replacements	486410	80165	23849	111874	19599	4760	66172	5801	5173
*Z*	−1.209	−0.582	−0.777	−3.852	−3.331	−0.810	0.005	1.123	−0.793
sim. Mean	364287.2	68848	19463.302	53970.1	10251	3850.157	66281.4	9163	4232
sim. *SD*	100984.6	19416	5640.221	15031.06	2805	1122.994	19671.7	2992	1185
Method	R1	R1	R1	R1	R1	R1	R1	R1	R1
Clumping									
Index	2.918	3.309	2.546	2.314	1.911	2.484	2.181	3.857	1.434
*p*	**<.001**	**<.001**	**<.001**	**<.001**	**.001**	**<.001**	**<.001**	**<.001**	**.008**
	Quasi‐Clementsian	Random	Random	Clementsian	Clementsian	Random	Clumped species loss	Random	Random

**Figure 5:**
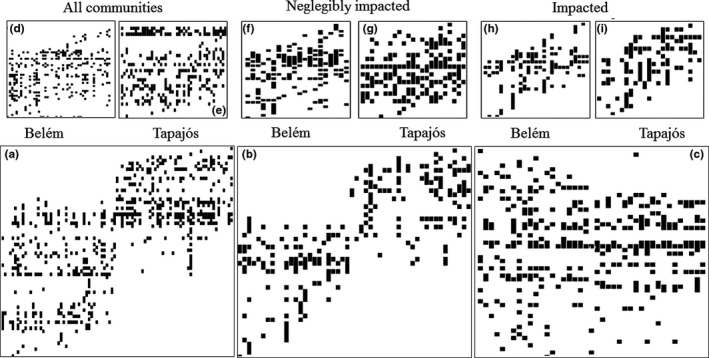
Ordination of the composition of Zygoptera communities in Amazonian streams. Horizontally represent the occurrence of species and vertically represent the spatial filter (principal coordinate analysis of neighbor matrices—PCNM1). (a) All 93 communities regardless of environmental conservation, (b) only the communities of the 43 streams negligibly impacted, (c) only the communities of the 50 streams impacted, (d) all 47 communities from Belém area of endemism, regardless of environmental conservation, (e) all 46 communities from Tapajós area of endemism, regardless of environmental conservation, (f) only 20 communities negligibly impacted streams of Belém area of endemism, (g) only 23 communities negligibly impacted streams Tapajós area of endemism, (h) only 27 communities impacted streams of Belém area of endemism, and (i) only 23 communities impacted streams of Tapajós area of endemism

When we analyzed the regions separately, the impacted streams of both regions (Tapajos and Belem areas of endemism) presented a random pattern of coherence. When the negligibly impacted streams of Tapajos area of endemism were added to the analysis, the random pattern was also found. However, when we analyzed the negligibly impacted streams of the Belem area of endemism, the pattern was Clementsian, with significantly positive coherence, turnover, and clumping (Table 2), with a similar (quasi‐Clementsian) pattern being found when all the communities (negligibly impacted and impacted streams) were analyzed together (Figure 5).

The patterns (Clementsian and quasi‐Clementsian) found in most of the metacommunities associated with negligibly impacted streams, and all communities, irrespective of region or environmental integrity, were closely related to the biogeographic configuration (areas of endemism). This pattern was particularly strong among the negligibly impacted stream communities, which differed greatly in their species composition between regions (PERMANOVA, pseudo *F* = 10.541_(1,96);_ *p* = .001). The patterns of clumped and random species loss observed in all the impacted streams indicate that environmental changes have caused changes in the elements of metacommunity structure. Evidence of these changes can be seen in the homogenization of communities in impacted streams compared to communities in negligibly impacted streams (PERMDISP, pseudo *F* = 67.202 _(1,96);_ *p* = .001) (Figure 5c).

## 4 Discussion

**Figure 6:**
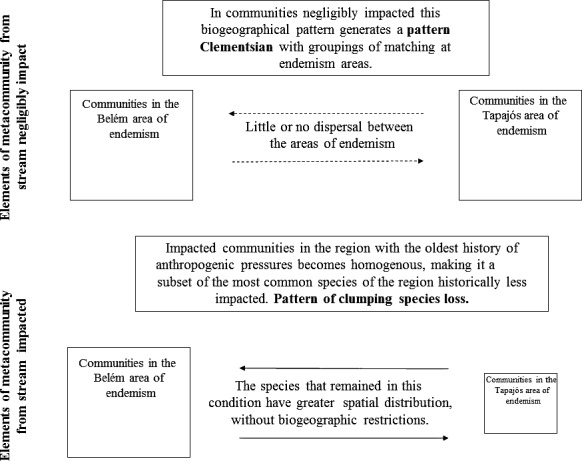
Graphical model representing the main results of the metacommunities standards. Streams with little change a limiting dispersion (dotted arrows) and communities have distinct compositions between the two biogeographic regions (pattern Clementsian). The second result demonstrates the nested pattern found between regions whereas only impacted communities, this dispersion is not limiting prospects (unbroken lines and arrows) (pattern clumped species loss)

Our hypothesis that communities at sites with less impact would present a Clementsian pattern, due to the biogeographic distribution of the species in the areas of endemism, was corroborated. The Clementsian pattern of the more negligibly impacted sites reflects the biogeographic configuration of the areas of endemism (Juen and de Marco, 2012). By contrast, the evidence of clumped species loss in the case of the impacted streams reflects the changes of these communities through the loss of zygopteran species (Oliveira‐Junior et al., 2015), principally in the Tapajós area of endemism, where there is a more extensive history of anthropogenic impact (Gardner et al., 2013). In this region, in fact, even the communities of the negligibly impacted streams presented a random pattern (Figure 6), giving indications that besides the intensity (negligibly impacted or impacted), the historical frequency of the alterations of the regions has also been an important process for the present communities.

The intensity of the impacts on the landscape is responsible for major changes in the patterns of species diversity (Gutiérrez‐Cánovas, Millán, Velasco, Vaughan, & Ormerod, 2013). In addition, the impacted streams of the Santarém region (Tapajós area of endemism) presented a subset of the species found in the communities of the impacted streams of Paragominas region (Belem area of endemism) (see Figure 5c). Comparing the same regions, Gardner et al. (2013) also found a lower taxonomic richness of bees, beetles, ephemeropterans, ants, heteropterans, plecopterans, odonates, and tricopterans in Santarém. These authors comment that whereas Santarém has been densely populated by farming communities of pre‐Columbian civilizations since 1661, Paragominas was sparsely populated until the 1980s, when the logging industry advanced into the region. Thus, the longer history of disturbance in the Santarém is the probable cause of the greater homogeneity of the biota of this region. These results reinforce the idea that the random structure indicates that species are not structured by responses to a common environmental gradient. It does not mean that there is no structure or that environment is not important, only that the responses may not be idiosyncratic along environmental gradients (Rodrigues et al., 2016) with different disturbance intensities (Petraitis, Latham, & Niesenbaum, 1989).

One fundamental variable in metacommunity analysis is the spatial scale of the area analyzed, given that different mechanisms may operate at each scale leading to distinct patterns (local or regional) of distribution (Presley et al., 2010). This occurs because, on a smaller scale, environmental gradients and spatial processes have different effects on the distribution of species (Presley & Willig, 2010). According to the theory of isolation by rivers (Wallace 1854), for example, higher levels of dispersal are expected between the communities found in the same areas of endemism (Juen and de Marco, 2012). Given this, when we analyze small‐scale patterns of elements of metacommunity structures, that is, within areas of endemism, the spatial component may be less important, as found by Juen & De Marco (2011) in communities in Amazonian streams. The random patterns observed at this scale may in fact be related to the variation in the timing and the magnitude of the environmental impacts that are or were dynamic in these landscapes, either historically, as discussed by Gardner et al. (2013), or currently (see Leal et al., 2016). Both these studies focused on the same areas analyzed in the present study. The Clementsian patterns, found in most of the negligibly impacted stream communities and at the broader spatial scale, may be structured by biogeographic processes, as observed in the bat communities of Caribbean islands (Presley & Willig, 2010), or in communities affected by major environmental variation, such as that found in the tropical desert climate ecotone in Mexico (López‐González, Presley, Lozano, Stevens, & Higgins, 2012). In this case, the Clementsian pattern may be related to biogeographic features and/or environmental variations, as well as the historical factors that contribute to the spatial distribution of the species (Heino, Soininen, Alahuhta, Lappalainen, & Virtanen, 2015). Given this, we believe, on a large scale, the Clementsian pattern is related to the historical process of isolation of communities generated by the emergence of large rivers, making their areas of endemism distinct biogeographic units for Zygoptera communities in Amazonia (Juen and de Marco, 2012). However, when we consider the impacted sites only, the effect of environmental gradients on the communities is clear, as referred to in the species‐sorting perspective (Henriques‐Silva et al. 2013). This mechanism is very important for the distribution of Odonata, mostly at local scale, being mainly determined by gradients of human impact, such as those induced by land use changes, where generalist species are favored in altered habitats (De Marco et al., 2015).

There is much evidence to show that environmental filter is the main mechanisms for the structure of aquatic communities, especially when you consider small spatial scale (Cottenie, 2005; Van der Gucht et al., 2007; Mykrä et al., 2007, Heino, Nokela, et al., 2015); however, whereas in large spatial scale aquatic communities have a strong relationship with biogeographic units (such as the water catchment area or areas of endemism), and with that the communities have high values of beta diversity along the landscape (explained mainly by turnover), what generates Clementsian patterns along the landscape (Heino et al. 2016).

The interpretation of changes in the patterns of metacommunity structures is an important step in the analysis of the impact of environmental disturbances on natural communities. Our results show that the natural elements of metacommunity structures are altered due to environmental pressures that interfere directly on the coexistence of species, changing the rules of community assembly. Understanding the mechanisms that trigger these changes is an important issue for conservation, because they can help to create mitigating measures of the impacts of environmental changes on communities and so should be expanded in other studies using other taxonomic groups in tropical and temperate systems, and, wherever possible, at multiple spatial scales.

## Conflict of Interest

None declared.
