# Supplementary material for: Elements of metacommunity structure in Amazonian Zygoptera among streams under different spatial scales and environmental conditions
Source: Ecol Evol. 2017 Mar 31;7(9):3190–200. doi: 10.1002/ece3.2849 (PMC5415516; doi:10.1002/ece3.2849)
Supplement: Supplementary file 1 [file ECE3-7-3190-s001.doc]

**Table S1.** Descriptors characteristics of the environmental conditions of the streams. Table adapted from Nessimian et al. (2008).

| **Characteristic** | **Condition** | **Score** |
| --- | --- | --- |
| 1- Land use pattern beyond the riparian zone | Primary continue forest/100 ha fragment/10 ha fragment | 6 |
|  | Cecropia secondary forest/mixed secondary forest | 5 |
|  | Vismia secondary forest | 4 |
|  | Pasture | 3 |
|  | Perennial crops | 2 |
|  | Short-cycle crops/exposed soil | 1 |
| 2- Width of riparian forest | Continuous forest | 6 |
|  | Forest width between 30 and 100 m | 5 |
|  | Forest width between 5 and 30 m | 4 |
|  | Forest width between 1 and 5 m | 3 |
|  | Riparian forest absent, but some shrub species and pioneer trees | 2 |
|  | Riparian forest and shrub vegetation absent | 1 |
| 3- Completeness of riparian forest | Riparian forest intact without breaks in vegetation | 4 |
|  | Breaks occurring at intervals of < 50 m | 3 |
|  | Breaks frequent with gullies and scars at every 50 m | 2 |
|  | Deeply scarred with gullies all along its length | 1 |
| 4- Vegetation of riparian zone within 10 m of channel | More than 90% plant density by non-pioneer trees or shrubs | 4 |
|  | Mixed pioneer species and mature trees | 3 |
|  | Mixed grasses and sparse pioneer trees and shrubs | 2 |
|  | Grasses and few tree shrubs | 1 |
| 5- Retention devices | Channel with rocks and/or old logs ﬁrmly set in place | 4 |
|  | Rocks and/or logs present but backﬁlled with sediment | 3 |
|  | Retention devices loose, moving with ﬂoods | 2 |
|  | Channel of loose sandy silt, few channel obstructions | 1 |
| 6- Channel sediments | Little or no channel enlargement resulting from sediment accumulation | 4 |
|  | Some gravel bars of coarse stones and little silt | 3 |
|  | Sediment bars of rocks, sand and silt common | 2 |
|  | Channel divided into braids or stream channel corrected | 1 |
| 7- Bank structure | Banks inconspicuous | 5 |
|  | Banks stable, with rock and soil held ﬁrmly by grasses, shrubs, or tree roots | 4 |
|  | Banks ﬁrm but loosely held by grasses and shrubs | 3 |
|  | Banks of loose soil held by a sparse layer of grass and shrubs | 2 |
|  | Banks unstable, easily disturbed, with loose soil or sand | 1 |
| 8- Bank undercutting | Little, not evident or restricted to areas with tree root support | 4 |
|  | Cutting only on curves and at constrictions | 3 |
|  | Cutting frequent, undercutting of banks and roots | 2 |
|  | Severe cutting along channel, banks falling in | 1 |

| **Continued** |  |  |
| --- | --- | --- |
| **Characteristic** | **Condition** | **Score** |
| 9- Stream bottom | Stone bottom of several sizes packed together, interstices obvious | 4 |
|  | Stone bottom easily moved, with little silt | 3 |
|  | Bottom of silt, gravel, and sand, stable in some places | 2 |
|  | Uniform bottom of sand and silt loosely held together, stony substrate absent | 1 |
| 10- Rifﬂes and pools, or meanders | Distinct, occurring at intervals of 5-79 the stream width | 4 |
|  | Irregularly spaced | 3 |
|  | Long pools separating short rifﬂes, meanders absent | 2 |
|  | Meanders and rifﬂe/pools absent or stream corrected | 1 |
| 11- Aquatic vegetation | When present, consists of moss and patches of algae | 4 |
|  | Algae dominant in pools, vascular plants along edge | 3 |
|  | Algal mats present, some vascular plants, few mosses | 2 |
|  | Algal mats cover bottom, vascular plants dominate channel | 1 |
| 12- Detritus | Mainly consisting of leaves and wood, without sediment | 5 |
|  | Mainly consisting of leaves and wood, with sediment | 4 |
|  | Few leaves and wood, ﬁne organic debris, with sediment | 3 |
|  | No leaves or woody debris, coarse and ﬁne organic matter, with sediment | 2 |
|  | Fine anaerobic sediment, no coarse debris | 1 |

**Table S2.** Checklist of species by environments from Belém area of endemism and Tapajós area of endemism.

| **Taxa** | **Belém** | | **Tapajós** | |
| --- | --- | --- | --- | --- |
|  | **Negligible impacted** | **Impacted** | **Negligible impacted** | **Impacted** |
| *Acanthagrion adustum* Williamson, 1916 |  | x |  |  |
| *Acanthagrion aepiolum* Tennessen, 2004 | x | x |  |  |
| *Acanthagrion apicale* Selys, 1876 | x | x |  | x |
| *Acanthagrion ascendens* Calvert, 1909 | x | x |  |  |
| *Acanthagrion jessei* Leonard, 1977 |  | x |  |  |
| *Acanthagrion kennedii* Williamson 1916 | x | x | x | x |
| *Acanthagrion rubrifrons* Leonard, 1977 | x |  |  |  |
| *Acanthallagma luteum* Williamson and Williamson, 1924 | x | x |  |  |
| *Argia fumigata* Hagen in Selys, 1865 | x | x | x | x |
| *Argia infumata* Selys, 1865 | x |  | x | x |
| *Argia insipida* Hagen in Selys, 1865 | x |  |  |  |
| *Argia mollis* Hagen in Selys, 1865 | x | x |  |  |
| *Argia reclusa* Selys, 1865 | x |  |  |  |
| *Argia smithiana* Calvert, 1909 | x |  |  |  |
| *Argia thespis* Hagen in Selys, 1865 | x | x |  |  |
| *Argia tinctipennis* Selys, 1865 | x | x | x | x |
| *Argia tupi* Calvert, 1909 | x |  |  |  |
| *Chalcopteryx rutilans* (Rambur, 1842) | x |  | x | x |
| *Dicterias atrosanguinea* Selys, 1853 | x |  | x | x |
| *Epipleoneura capilliformis* (Selys, 1886) | x |  | x | x |
| *Epipleoneura fuscaenea* Williamson, 1915 |  | x |  |  |
| *Epipleoneura metallica* Rácenis, 1955 | x | x |  |  |
| *Epipleoneura westfalli* Machado, 1986 | x | x |  |  |
| *Heliocharis amazona* Selys, 1853 | x | x | x |  |
| *Hetaerina auripennis* Burmeister, 1839 | x | x |  |  |
| *Hetaerina sanguinea* Selys, 1853 |  | x | x | x |
| *Heteragrion aurantiacum* Selys, 1862 | x | x |  |  |
| *Heteragrion icterops* Selys, 1862 | x | x | x | x |
| *Ischnura capreolus* (Hagen, 1861) |  | x |  |  |
| *Mecistogaster linearis* (Fabricius, 1776) | x |  | x |  |
| *Mnesarete aenea* (Selys, 1853) | x | x | x | x |
| *Mnesarete cupraea* (Selys, 1853) | x |  |  |  |
| *Mnesarete williamsoni* Garrison, 2006 | x | x |  |  |
| *Neoneura denticulata* Williamson, 1917 | x | x |  |  |

| **Continuation** | | | | | |
| --- | --- | --- | --- | --- | --- |
| **Taxa** | **Belém** | | | **Tapajós** | |
|  | **Negligible impacted** | | **Impacted** | **Negligible impacted** | **Impacted** |
| *Neoneura bilinearis* Rácenis, 1953 | |  | x |  |  |
| *Neoneura joana* Williamson, 1917 | |  | x |  |  |
| *Neoneura luzmarina* Marmels, 1989 | | x | x | x | x |
| *Neoneura rubriventris* Selys, 1860 | | x | x |  |  |
| *Oxystigma petiolatum* (Selys, 1862) | | x |  |  |  |
| *Perilestes kahli* Williamson & Williamson, 1924 | | x |  |  |  |
| *Perilestes solutus* Williamson & Williamson, 1924 | | x |  |  |  |
| *Protoneura tenuis* Selys, 1860 | | x |  | x |  |
| *Psaironeura tenuissima* (Selys, 1886) | | x |  | x | x |
| *Telebasis sanguinalis* Calvert, 1909 | |  | x |  |  |
| *Tigriagrion aurantinigrum* Calvert, 1909 | | x | x |  |  |
| *Argia chapadae* Calvert, 1909 | |  |  |  | x |
| *Argia oculata* Selys, 1865 | |  |  | x | x |
| *Argia euphorbia* Fraser, 1946 | |  |  | x | x |
| *Chalcopteryx radians* Ris, 1914 | |  |  | x | x |
| *Epipleoneura haroldoi* Santos, 1964 | |  |  | x | x |
| *Epipleoneura pereirai* Machado, 1964 | |  |  | x |  |
| *Epipleoneura spatulata* Rácenis, 1960 | |  |  |  | x |
| *Hetaerina indeprensa* Garrison, 1990 | |  |  | x | x |
| *Hetaerina* *rosea* | |  |  | x |  |
| *Mnesarete smaragdina* (Selys, 1869) | |  |  | x | x |
| *Oxystigma williamsoni* Geijskes, 1976 | |  |  | x | x |
| *Perilestes attenuatus* Selys, 1886 | |  |  | x |  |
| *Phasmoneura exigua* (Selys, 1886) | |  |  | x |  |
| *Telebasis* sp. nov. | |  |  | x | x |
| *Acanthagrion* sp.1 | |  | x |  |  |
| *Argia* sp.1 | | x | x |  |  |
| *Argia* sp.2 | |  | x |  |  |
| *Argia* sp.3 | | x | x |  |  |
| *Argia* sp.4 | | x |  |  |  |
| *Argia* sp.5 | | x | x |  |  |
| *Argia* sp.6 | |  | x |  |  |
| *Argia* sp.7 | | x |  |  |  |
| *Argia* sp.8 | | x | x |  |  |
| *Argia* sp. | |  |  | x | x |
| *Heteragrion* sp.nov. | | x |  | x | x |
| **Ocorrence (%)** | | **64.28** | **51.42** | **35.71** | **35.71** |

**Figure S3.** Equations of Habitat Integrity Index, adapted from Nessimian et al. (2008). A= Equation 1, ponders the weight of the items within each feature examined, and B = 2 equation, calculates the index whereas the values obtained within 12 characteristics weighted (Equation 1). *p*_i=_ value weighted of characteristic; *a*_o_= value observed; *a*_m_= Maximum value; HII= Habitat Index Integrity.

| A) | B) |
| --- | --- |
| 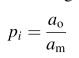 | 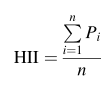 |
